# Can the application of the international health regulations to antimicrobial resistance events help to preserve antimicrobials?

**DOI:** 10.1186/1753-6561-5-S6-O40

**Published:** 2011-06-29

**Authors:** D Wernli, T Haustein, J Conly, S Harbarth

**Affiliations:** 1 Division of International and Humanitarian Medicine, University of Geneva Hospitals and Faculty of Medicine, Geneva, Switzerland; 2 Infection Control Program, University of Geneva Hospitals and Faculty of Medicine, Geneva, Switzerland

## Introduction / objectives

The global threat of antimicrobial resistance (AMR) needs to be addressed urgently. The global surveillance of AMR pathogens is patchy and limited by financial and technical constraints. Without an early-warning system, the emergence and spread of AMR often goes unnoticed until a given strain has become endemic.

## Methods

Using the example of carbapenem-resistant *Enterobacteriaceae* (CRE), we analyzed the potential role of the International Health Regulations (IHR), a legally binding agreement between 194 States Parties, whose aim is “to prevent, protect against, control and provide a public health response to the international spread of disease” with respect to AMR and assess whether selected CRE events fulfil the four criteria of Annex 2 of the IHR.

## Results

Certain events marking the emergence and international spread of KPC and NDM-1-producing CRE fulfil the criteria for notifiability to WHO. This can be extrapolated to other types of AMR. At the same time, ambiguities in Annex 2 and limited specific WHO guidance may make notification decisions a matter of debate. Obstacles for the application of the IHR to AMR include a lack of capacities within WHO.

## Conclusion

The global threat posed by the spread of AMR requires a coordinated international response. Recognizing the applicability of the IHR to AMR could serve as a “wake-up call” and obligate WHO and States Parties to strengthen surveillance and response, which could in turn contribute to containing the spread of AMR and preserve the efficacy of antimicrobials. Although States Parties and WHO share a collective responsibility in the process, WHO must clearly delineate its position regarding AMR and the intended role of the IHR in this context.

## Disclosure of interest

None declared.

